# Supplementary material for: Revealing hidden interlayer excitons in 2D bilayers via hybrid molecular gating
Source: Nat Commun. 2025 Nov 10;16:9893. doi: 10.1038/s41467-025-65431-6 (PMC12603254; doi:10.1038/s41467-025-65431-6)
Supplement: Supplementary file 1 — Supplementary Information [file 41467_2025_65431_MOESM1_ESM.pdf]

Supplementary Information for

"Revealing hidden interlayer excitons in 2D bilayers via hybrid molecular gating"

S. Kovalchuk[1], K. Greben[1], A. M. Kumar[1], S. Pessel[1], J. Soyka[2], Q. Cao[2], K. Watanabe[3], T. Taniguchi[3], D. Christiansen[4], M. Selig[4], A. Knorr[4], S. Eigler[2], K.I. Bolotin[1*]

[1] Physics Department, Freie Universität Berlin, Germany
[2] Institute of Chemistry and Biochemistry, Freie Universität Berlin, Germany
[3] National Institute for Materials Science, Tsukuba, Japan
[4] Physics Department, Technische Universität Berlin, Germany

Supplementary note S1: 3-capacitor electrostatic model

We developed a simple model based on previous work[1] to evaluate the carrier densities as well as electric fields inside a bilayer TMD. In this model, we treat the bilayer as a two decoupled and closely spaced TMD monolayers. The TMD is on top of the SiO_2/Si substrate (biased V_G, capacitance C_G vs. TMD). There is a charge density inside SiO_2 affecting the TMD – it is modelled as additional potential V_0 applied to the bottom gate. The top molecular layer is modeled as a top gate electrode kept at potential V_{LUMO}. By equating the potentials between the layers, we get the following equations:

$$eV_{LUMO} = E_F^{TOP} + \frac{e^2 n_{TOP}}{C_{mol}} + \frac{eC_{BL}}{C_{mol}}(E_F^{TOP} - E_F^{BOT}) \qquad (1)$$

$$eV_G + eV_0 = E_F^{BOT} + \frac{e^2 n_{BOT}}{C_G} - \frac{eC_{BL}}{C_G}(E_F^{TOP} - E_F^{BOT}) \qquad (2)$$

$$n_{TOP} = \int_{-\infty}^{E_F^{TOP}} g(\varepsilon)\cdot f(\varepsilon)\cdot d\varepsilon \qquad (3)$$

$$n_{BOT} = \int_{-\infty}^{E_F^{BOT}} g(\varepsilon)\cdot f(\varepsilon)\cdot d\varepsilon \qquad (4)$$

Here E_F^{TOP}, E_F^{BOT} are Fermi energies relative to the middle of the bandgap in top/bottom TMD layers of a bilayer; n_{TOP}, n_{BOT} are carrier densities inside respective layers; C_{TOP}, C_{BOT} are per-area capacitances between top (bottom) gate electrode and the TMD; C_{BL} is the capacitance between the TMD layers; $g(\varepsilon) = g_s g_v m^*/2\pi\hbar^2$ is density of states (DOS) function for the 2D gas inside each TMD layer treated independently; f(ε) is the Fermi-Dirac occupation function, approximated as a step function[2].

The field inside the TMD in this model:

$$F_Z = \frac{eC_{BL}}{\varepsilon_0 \varepsilon_{TMD}}\left(E_F^{TOP} - E_F^{BOT}\right) \qquad (5)$$

Inside the bandgap, we assume that $n_{TOP} = 0$, $n_{BOT} = 0$:

$$eC_{BL}(E_F^{TOP} - E_F^{BOT}) = (eV_{LUMO} - E_F^{TOP})C_{mol} \equiv \sigma_t \qquad (6)$$

$$eC_{BL}(E_F^{TOP} - E_F^{BOT}) = (E_F^{BOT} - eV_G)C_G - V_0 C_G \approx -eV_G C_G - \sigma_b \qquad (7)$$

Here $(eV_{LUMO} - E_F^{TOP})C_{mol}$ can be interpreted as a charge on top of the TMD, and the $\sigma_b = V_0 C_G$ is interpreted as a charge on the bottom interface of the TMD.

Combing eqns. 5-7, we get the equation 1 of the main text:

$$F_Z \approx \frac{1}{2\varepsilon_{TMD}}(\sigma_t - \sigma_b - eV_G C_G) \qquad (8)$$

Under the charge neutrality assumption $\sigma_t + \sigma_b + V_G C_G = 0$, it can be rewritten as:

$$F_Z \approx \frac{\varepsilon_{SIO2}}{\varepsilon_{TMD} d_{SIO2}}(V_G + V^0) \qquad (9)$$

Eqns. 8, 9 hold only for the case where E_F^{TOP}, E_F^{BOT} are inside if bandgap, and V_G is large enough to satisfy relation $E_F^{BOT} - eV_G \approx eV_G$.
Applying Eq. 9 to the device #2, shown in the Fig. 2a to calculate the maximum electric field:

$$F_Z^{max} = \frac{3.8}{6.8 \cdot 285\, nm}(90 + 86.6\, V) = 0.346\, V\, nm^{-1}.$$

The full solution of equations (1 - 4) shown in SI fig. 2 for devices #1, #3 was obtained using non-linear equations solver (optimize.fsolve python function from scipy module).

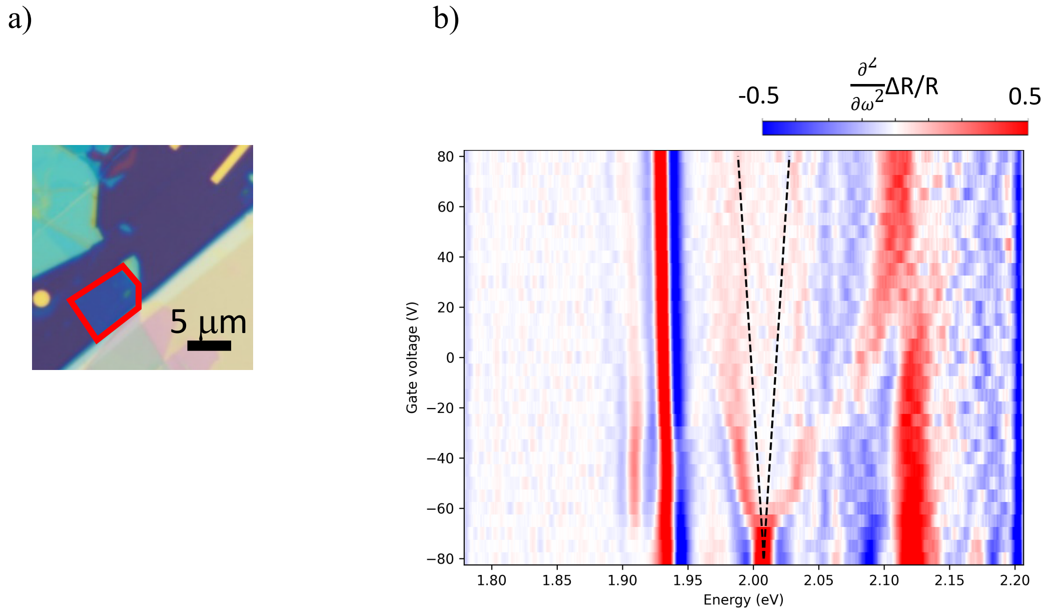

SI figure 1: (a) Microscopic image of device #1 **(b)** Second derivative of reflectivity contrast vs. V_G map for the same device, in a "clean" state. Note that there are no molecules on top of that device, and we observe the appearance of the trion peak at 1.92eV for V_G>-75V. In this case $\sigma_b = 5.9 \cdot 10^{12} \mathrm{cm}^{-2}$, found using known capacitance of the bottom gate. Dashed lines show the splitting of IX_1 calculated using the model in the text, assuming $\sigma_t = 0$. We note slight deviation of the experimentally measured position of IX_{1+} deviates from the predictions of that model. This could be due to interaction of the state with free carriers.

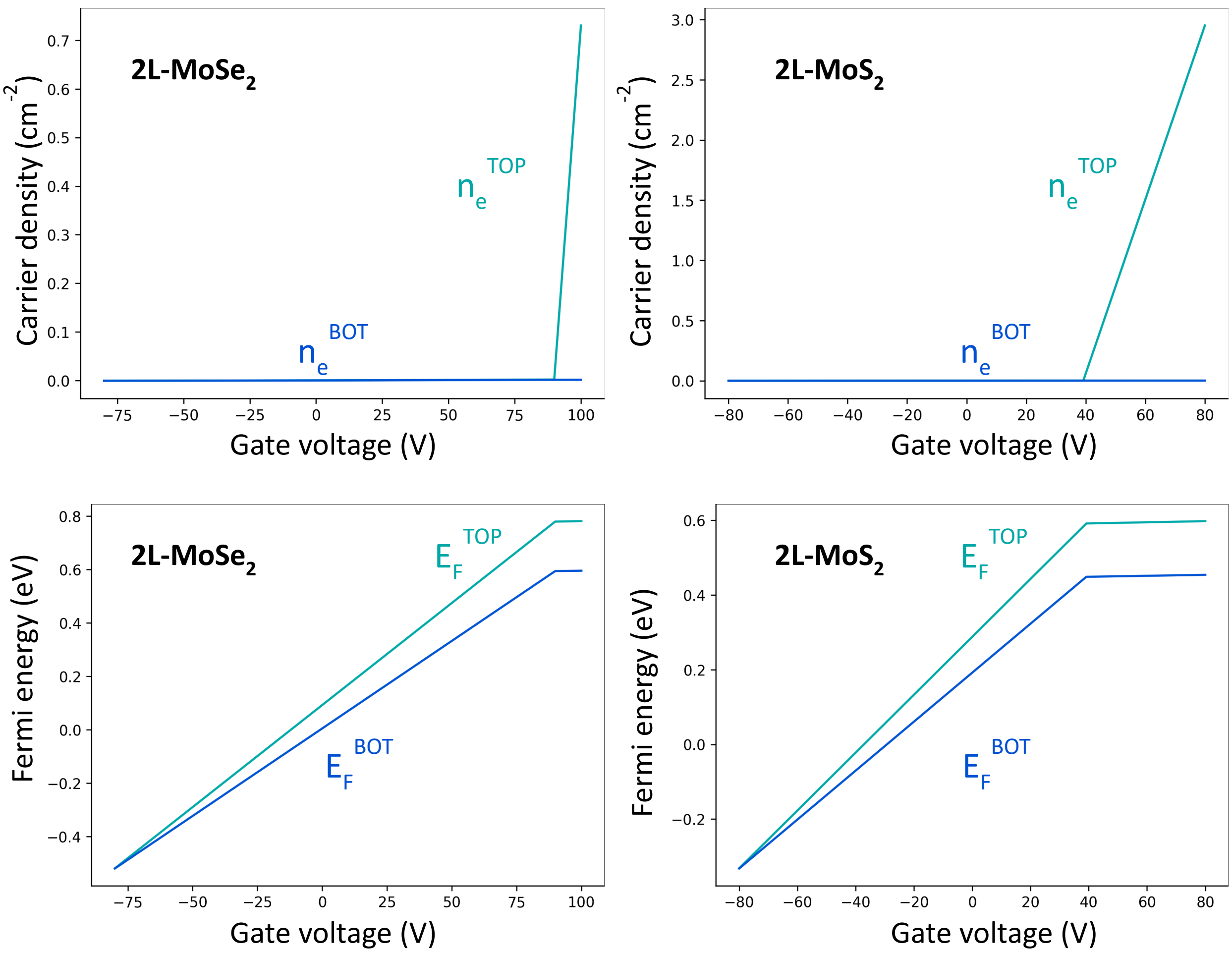

SI figure 2: Layer-resolved Fermi energies and carrier densities in 2D bilayer vs. V_G obtained by solving Eqs. (1-4) for the device #1 (2L-MoS_2/F_4TCNQ), and device #3 (2L-$MoSe_2$/F_4TCNQ), using parameters in SI table 1.

	2L-MoS_2	2L-$MoSe_2$
E_{VBM} (meV)	6158.5	5557.1
E_{CBM} (meV)	4373.7	3997.1
E_{BG} (meV)	1784.8	1560.0
E_{LUMO} (meV)	5300	5300
ε	6.8	7.9
C_{BL} (mF/m^2)	97	107.5
C_{mol} (mF/m^2)	17.9	17.9
C_G (mF/m^2)	0.118	0.118

SI table 1: Parameters used in modelling double gated-TMD devices. E_{VBM} and E_{CBM} taken from Ref. [3]. C_G calculated assuming the 285 nm thickness and 3.8 dielectric constant of the SiO_2 layer of the bottom gate. The parameter C_{mol} is found by matching the expected V_G at which the Fermi level enters into the conduction band in one of the layers to the experimental data monitoring the appearance of the trion peak. E_{LUMO} is taken from the Ref [4–6]; C_{mol} is lower than expected from the layer of charge with few angstrom separation from the surface. This could be a result of a low doping power of the molecule. Dielectric constants for two TMDs are taken from Ref. [7].

Supplementary note S2: Top and bottom molecular layers charge densities determination

The charge densities σ_t and σ_b are determined from optical spectroscopies of molecular gated devices vs. gate voltage. Two signatures that we follow, are: i) the gate voltage where $F_Z = 0$, signaled by zero splitting between IX_{1+} and IX_{1-}, and ii) the gate voltage where TMD Fermi energy enters conduction band, and we start observing trions.

First, the charge density inside SiO_2 traps σ_b, corresponds to the voltage where $F_Z = 0$, as can be seen from Eq. 9:

$$F_Z \approx \frac{\varepsilon_{SIO2}}{\varepsilon_{TMD} d_{SIO2}}(V_G + V^0)$$

In Fig. 2a in the main text, for instance, this happens at $V_G = -86.6$ V, determined by fitting of IX_{1+} and IX_{1-} resonances. This allows us to determine $V^0 = 86.6$ V, and corresponding $\sigma_\mathrm{b} = V^0 C_G = 6.4 \cdot 10^{12}\mathrm{cm}^{-2}$. It is considered to be constant within a single voltage sweep, and within a single evaporation. On the other hand, values for σ_b between different evaporations, as experiments demonstrate, can slightly change (SI Fig. 3).

To find σ_t, gate voltage dependent charge density inside molecules, we follow the second spectroscopic signature – appearance of the trions. As long as TMD is kept neutral, the charge conservation condition holds, and carrier density inside of the top molecular layer σ_t:

$$\sigma_t = -\sigma_b - V_G C_G$$

At the same time σ_t depends on the difference between the TMD Fermi energy (E_F) and the molecules' LUMO energy. In the model that we use (Eq. 6):

$$\sigma_t = C_{mol}(E_F - E_{LUMO})$$

here E_{LUMO} is the lowest unoccupied molecular orbital energy of the molecule, that is taken from a literature ($E_\mathrm{LUMO} = -5.3$ eV for F_4TCNQ) or the cyclic voltammetry measurement ($E_\mathrm{LUMO} = -5.94$ eV for CN6-CP, SI Fig. 12). C_{mol} is the effective geometrical capacitance of the molecular layer.

σ_t reaches its maximum value once E_F enters the conduction band, and becomes nearly static due to high DOS. Further increase of V_G mostly contributes towards charging TMD. Thus, experimentally measured voltage V_G where trions just start to appear determines E_F, σ_t^{max} and C_{mol}.

For different molecular coverages, the cut-off voltage changes, modeled by the changes of the effective capacitance C_{mol} and resulting σ_t^{max}. For the device #2, at the complete molecular coverage $\sigma_T^{max} = 13.08 \cdot 10^{12}\mathrm{cm}^{-2}$. For the device #1, one can see the evolution of the trion resonance peak with each evaporation in SI Fig. 3 and extracted σ_t^{max} in main text Fig. 1e. At the complete molecular coverage, we extract $C_\mathrm{mol} = 17.9$ mF/m^2, which corresponds to a molecule/TMD distance of ~1 nm.

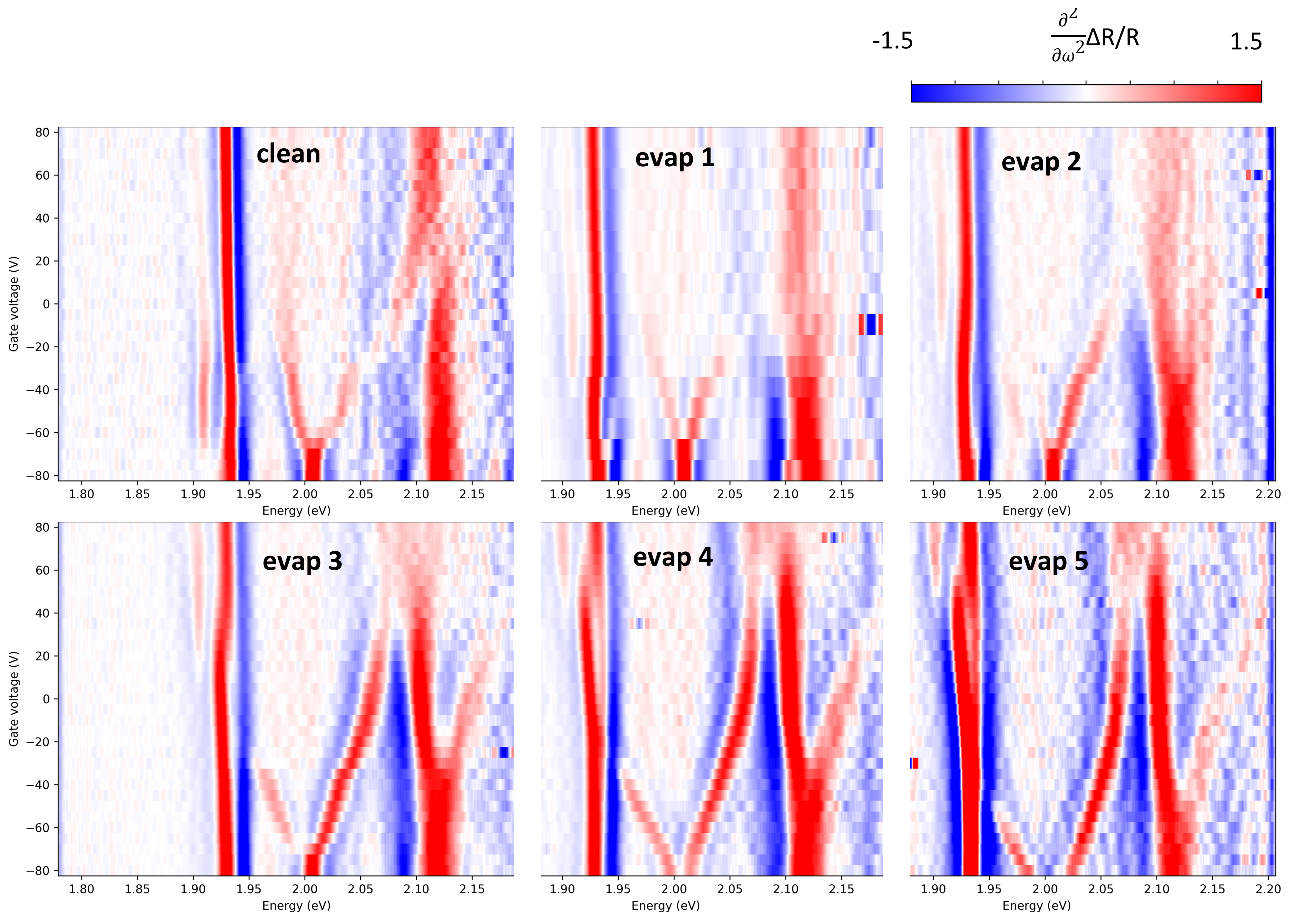

SI figure 3, F_4TCNQ/2L-MoS_2 device #1 at various evaporation stages: Second derivative of reflectivity contrast vs. V_G, for several successive evaporations of **F_4TCNQ**.

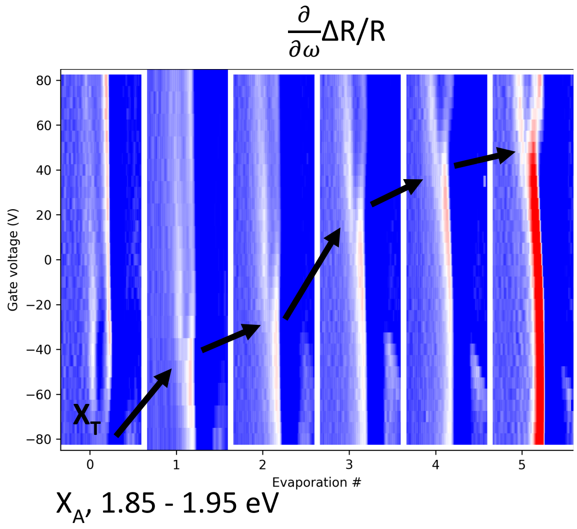

SI figure 4: Spectra from Fig. S3 zoomed in on the trion position region, 1.85 –1.95 eV. We observe that the gate voltage at which the trion peak appears changes for different evaporations signaling changes in the transferred carrier density between evaporations.

Supplementary note S3: Extraction of the dielectric function using Kramers-Kronig fitting model

To extract absorption from reflectivity contrast (R_C), we model the dielectric function of the TMD and the molecular layers using multiple Lorentzians, that according to Kramers-Kronig relations bind imaginary and real parts[8,9]:

$$\varepsilon(E) = \varepsilon_b + \sum \frac{a_i}{E_i^2 - E^2 - i \cdot \Gamma_i \cdot E}$$

Next, we use the transfer matrix method (TMM python module) to calculate the reflectivity contrast of the system and the difference between that contrast vs. experimental data. The non-linear fit routine is used to minimize that difference using the parameters of Lorentzians (amplitudes, positions, and widths for each resonance) and other free parameters. The voltage map of imaginary part of the dielectric constant and individual spectra fitted using this procedure are shown below:

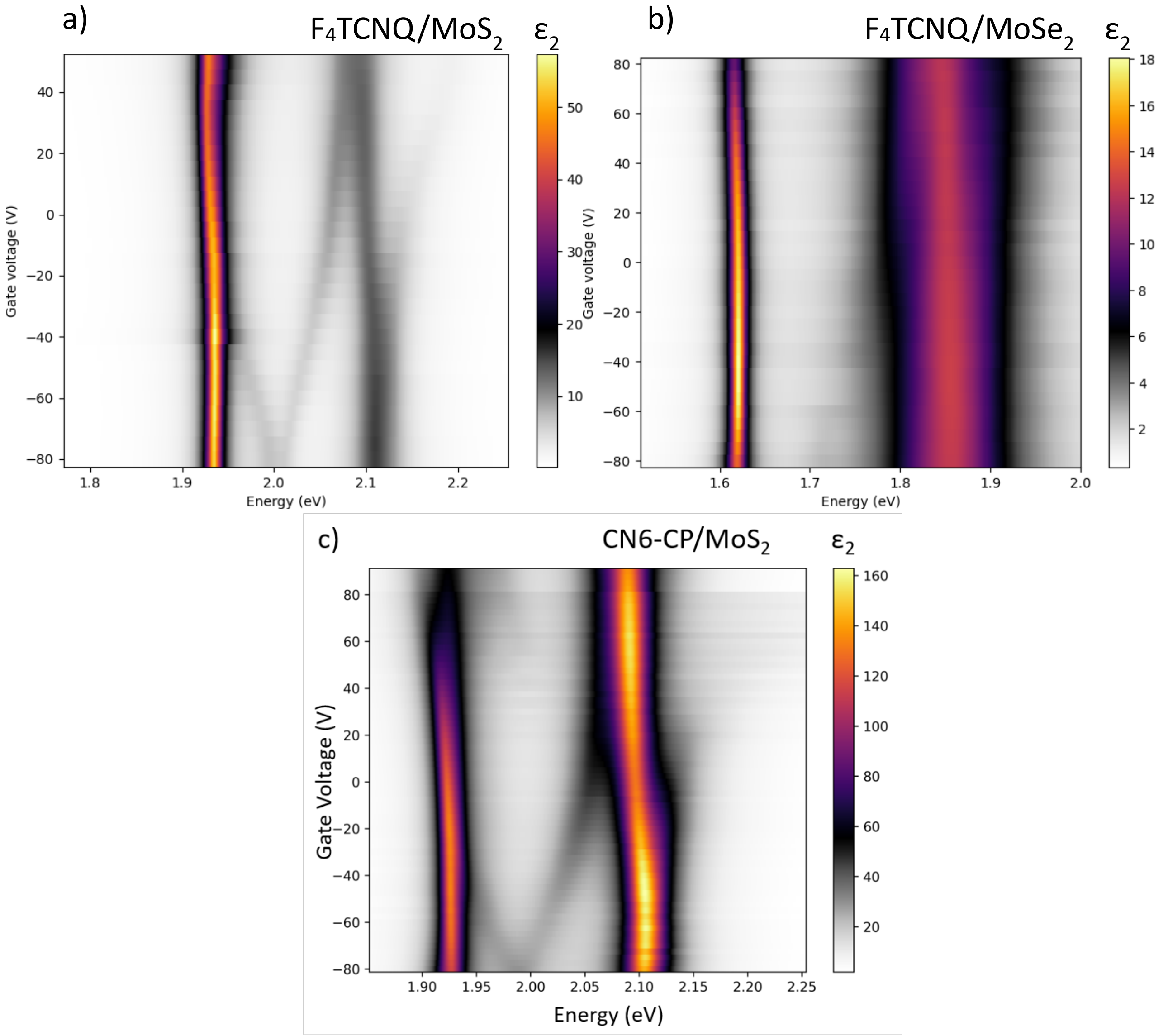

SI figure 5. Imaginary part of the dielectric function obtained using the fitting approach discussed above for: **(a)** MoS_2 device #1, with F_4TCNQ. **(b)** $MoSe_2$ device #3, with F_4TCNQ **(c)** MoS_2 device #2, with CN6-CP

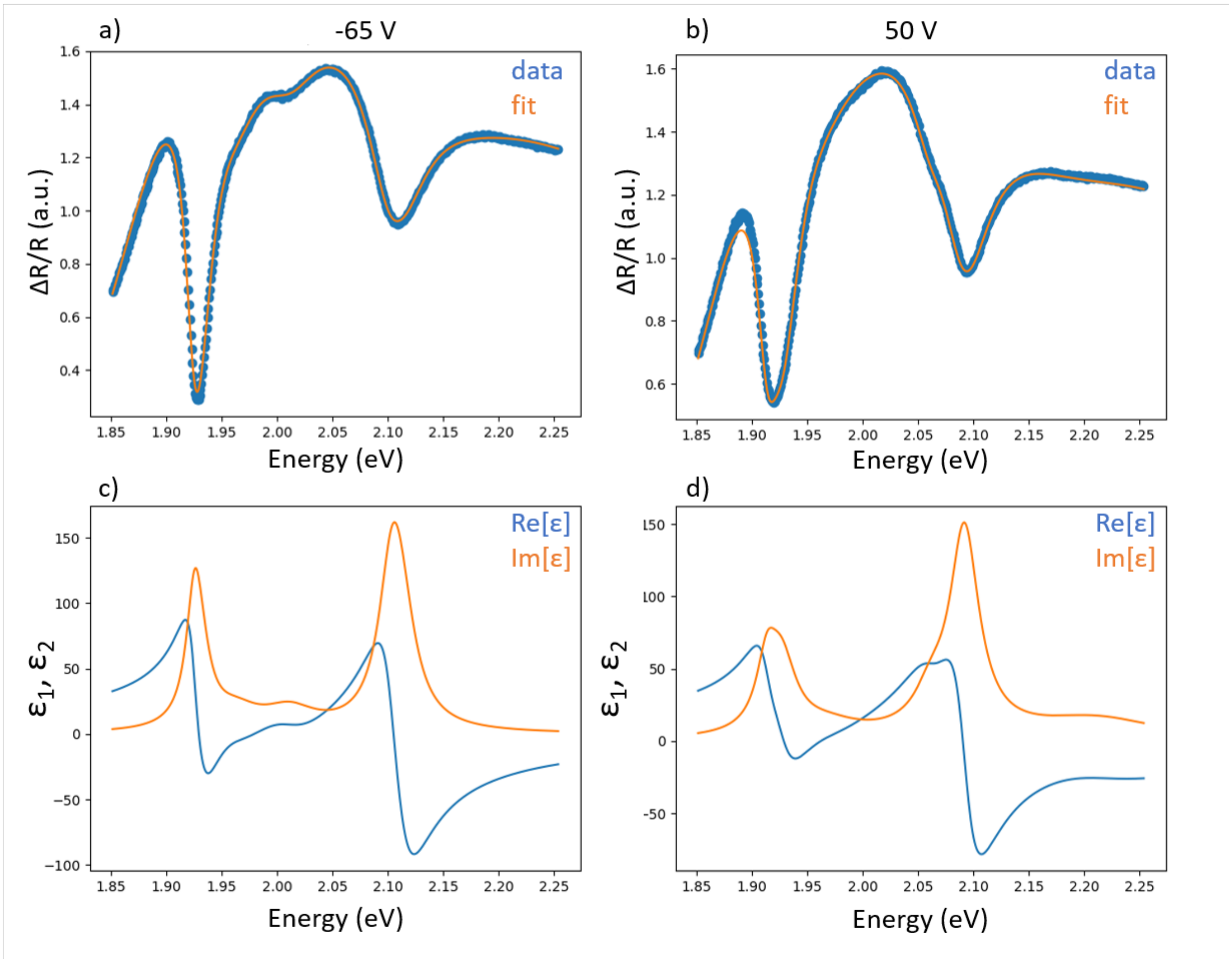

SI figure 5.2. (a, b) Reflectivity contrast and corresponding fit, at two different voltages for CN6CP/MoS_2 device #2. **(c, d)** Corresponding imaginary and real parts of the dielectric function of TMD.

Supplementary note S4: Extraction of the coupling strength parameter T and excitonic positions

First, we assume that when interexciton coupling is neglected, the energies of intralayer excitons (E_X') remain constant vs. field, while the energies of interlayer excitons (E_{IX}') are given by $E_{IX\pm}' = E_{IX}^{0'} \pm F_Z \times d_{BL}$. When the coupling is added, the excitonic energies are renormalized. A simple model based on the Bloch formalism gives the effect of interexcitonic coupling on the positions (E_X, E_{IX}) and oscillator strengths (P_X, P_{IX}) of the excitons[10] :

$$E_X, E_{IX} = \frac{E_X' + E_{IX}'}{2} \pm \sqrt{\frac{(E_X' - E_{IX}')^2}{4} + T^2} \quad (9)$$

$$P_X, P_{IX} = \frac{1}{2} \pm \frac{E_X' - E_{IX}'}{\sqrt{(E_X' - E_{IX}')^2 + T^2}} \quad (10)$$

Using the non-linear least square algorithm, we fit the predictions of that model to the extracted position of absorption peaks (SI Fig. 5) to the results the model (eq. 9).

First, in order to find unperturbed excitonic positions (E_X', E_{IX}'), tunneling strength (*T),* and the dipole moment (d_{BL}) we fit the model to the extracted absorption peak energies of X_B and IX_1. In this case in Eqs. (9, 10): $E_X' = X_B + \Delta X_B$, $E_{IX}' =$ IX1 $+ \Delta IX_1 \pm F_Z \times d_{BL}$. To fit the position of IX_2, we model another interlayer exciton that couples to X_A using the same Eqs. (9, 10): $E_X' = X_A + \Delta X_A$, $E_{IX}' = IX_2 + \Delta IX_2 \pm F_Z \times d_{BL}$, where d_{BL} and T are fitted together with the previous part. Results of the fits are shown in the main text Fig. 2a, Fig. 3c, and SI fig. 7. The accuracy of this fit depends on the possibility to resolve the shift and splitting of X_A. For the device #2, that reaches the highest electric field values, we add additional parameter β_Z – intralayer exciton polarizability, that introduces quadratic shift with electric filed[9,11] (details in SI Fig. 8).

	F_4TCNQ/2L-MoS_2	CN6-CP/2L-MoS_2	F_4TCNQ/2L-$MoSe_2$
X_A (eV)	1.935 ± 0.001	1.93 ± 0.001	1.621 ± 0.001
X_B (eV)	2.11 ± 0.001	2.104 ± 0.001	1.863 ± 0.002
IX_1 (eV)	2.003 ± 0.001	1.986 ± 0.002	1.715 ± 0.002
IX_2 (eV)	2.169 ± 0.003	2.146 ± 0.003	1.992 ± 0.014
ΔX_A (meV)	-7.3	-6.7	-5.5
ΔX_B (meV)	18.7	13.4	15.0
ΔIX_1 (meV)	-18.7	-13.4	-14.4
ΔIX_2 (meV)	7.3	6.7	6.0
$X_B - X_A$ (meV)	174.8	174.8	241.1
$IX_2 - IX_1$ (meV)	165.9	160	276.9
d_{BL} (e · nm)	0.60 ± 0.01	0.58 ± 0.01	0.65 ± 0.03
T (meV)	40.59 ± 0.43	37.58 ± 1.09	44.65 ± 2.16
V_0 (Volts)	82.2 ± 0.8	86.6 ± 1.2	80
σ_t^{max} ($10^{12} cm^{-2}$)	9.01 ± 0.06	13.01 ± 0.09	11.79 ± 0.3

SI table 2. Exciton energies without "Δ" denote observed energies of excitons at $F_Z = 0$ V nm^{-1}, fitted from reflectivity spectrum (SI note S3). Energies with "Δ" denote fitted energy shifts in absence of inter-excitons interactions, described above (SI note S4). Dipole moment d_{BL} and coupling strength T are also found from fitting to the model described coupling model.

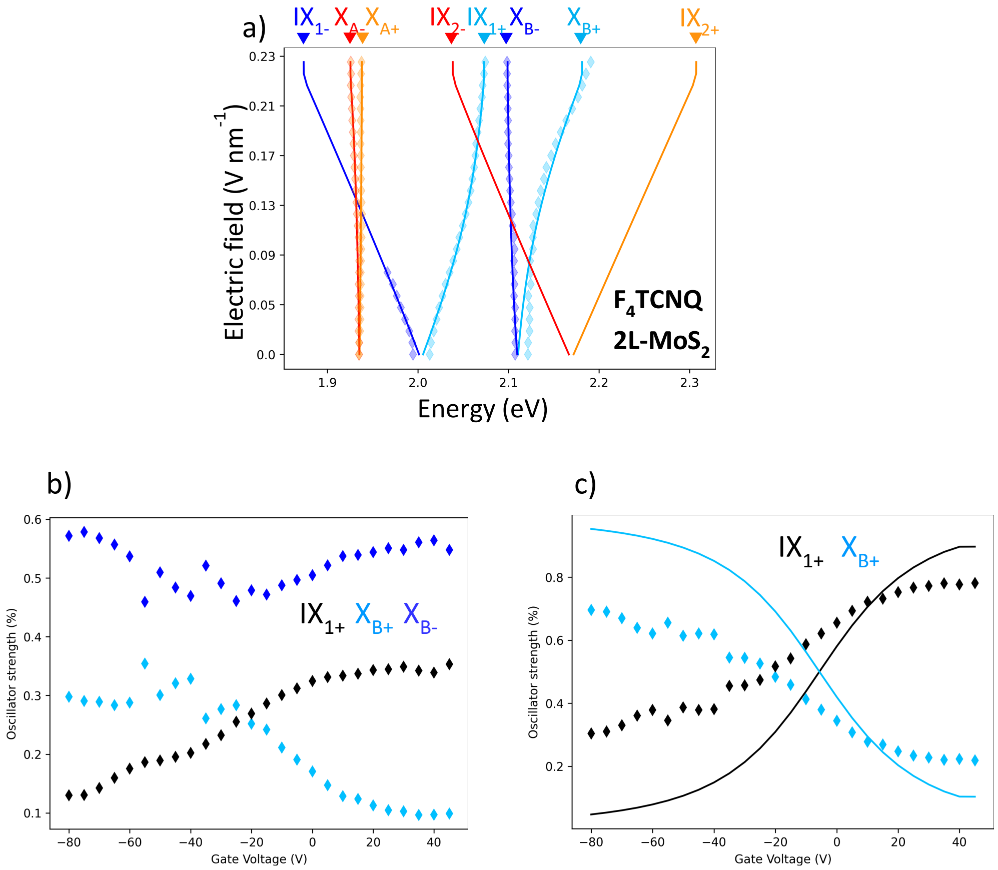

SI figure 6: (a) Positions of excitons extracted from the absorption map (points) vs. the results of the fit (lines) for device #1 $F_4TCNQ/2L\text{-}MoS_2$. **(b)** Oscillator strengths vs. V_G f or three excitons states. The data is extracted from the imaginary part of the dielectric function. Normalization is done relative to the sum of the oscillator strengths of these three states. X_{B+} and IX_{1+} show signature of coupling, as predicted by our model. **(c)** Fitted oscillator strength for two states (diamonds), as the model considers interaction only between two states. In this case we normalize oscillator strength to the sum of amplitudes of two peaks. Solid lines show result from the model, eq. 10.

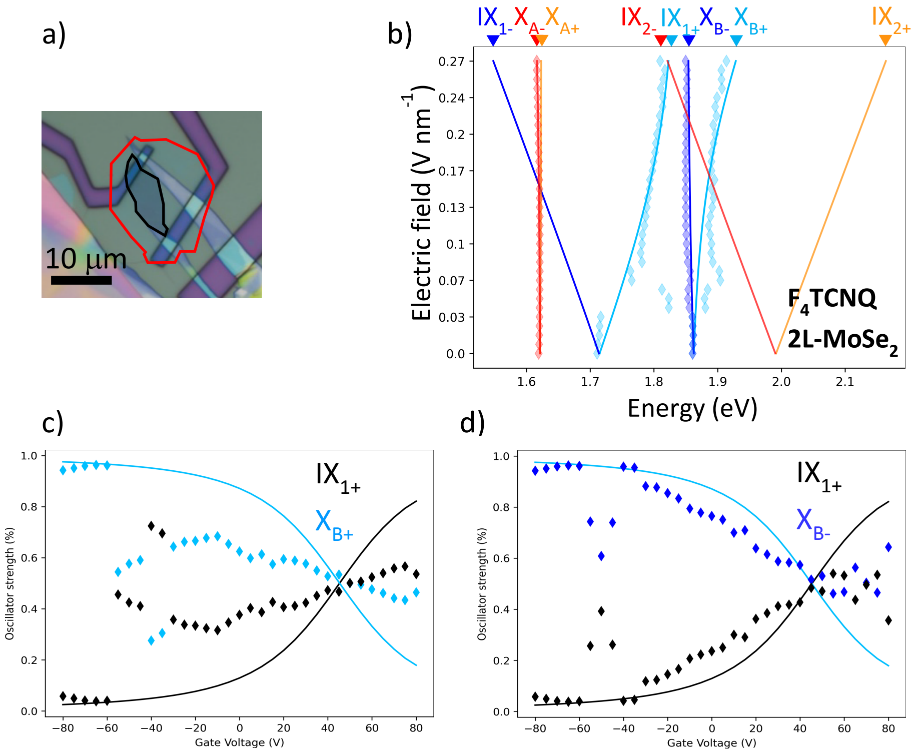

SI figure 7: (a) Microscope image of $F_4TCNQ/2L\text{-}MoSe_2$ device #3. **(b)** Positions of excitons extracted from the absorption map (points) vs. the results of the fit (lines). **(c)** Oscillator strengths for two excitonic states that are expected to be coupled, extracted from the dielectric function imaginary part amplitude, as a function of applied voltage. Normalization is done relative to the sum of the oscillator strengths of these states. Solid lines show theoretical model from eq. 10. **(d)** Same, but plotting comparison with X_{B-}. As two states X_{B+}, and X_{B-} are convoluted for the big part of the gate voltage range, oscillator strength can be uncertain between the two.

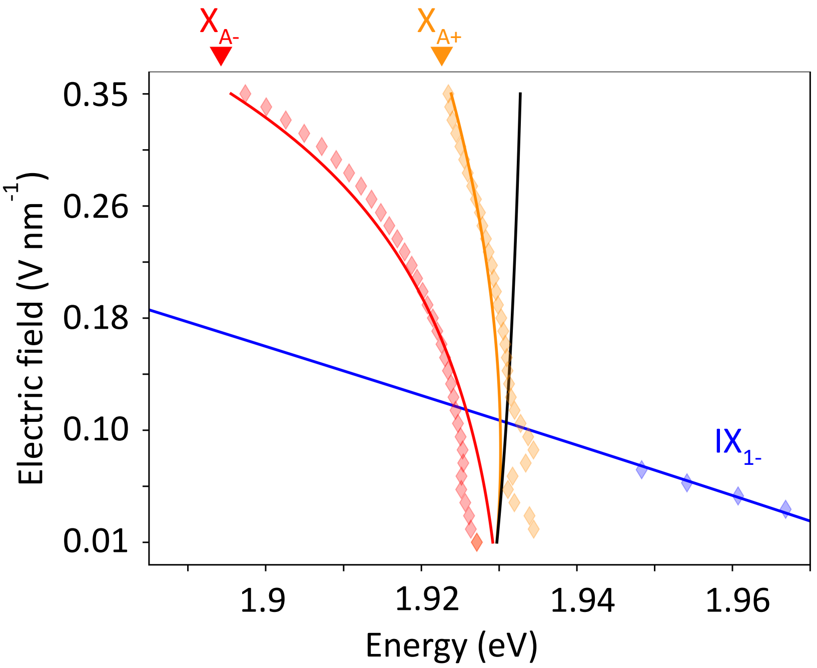

SI figure 8. MoS_2 device #1, with CN6-CP. Positions of excitons extracted from the absorption map (points) vs. the results of the fit to the model (lines). The orange line corresponds to the model that takes in account exciton polarizability before calculating coupling to interlayer exciton. The model for exciton polarizability follows[9,11]: $\Delta E = -\beta_Z F_Z^2$. It is applied to X_{A+}, X_{A-}, X_{B+} and X_{B-} excitons. When the exciton polarizability is ignored (black line), the electric field dependence for the X_{A+} shows an opposite trend. Fitted value for the β_Z is 3.59 ± 0.42 D nm V^{-1}.

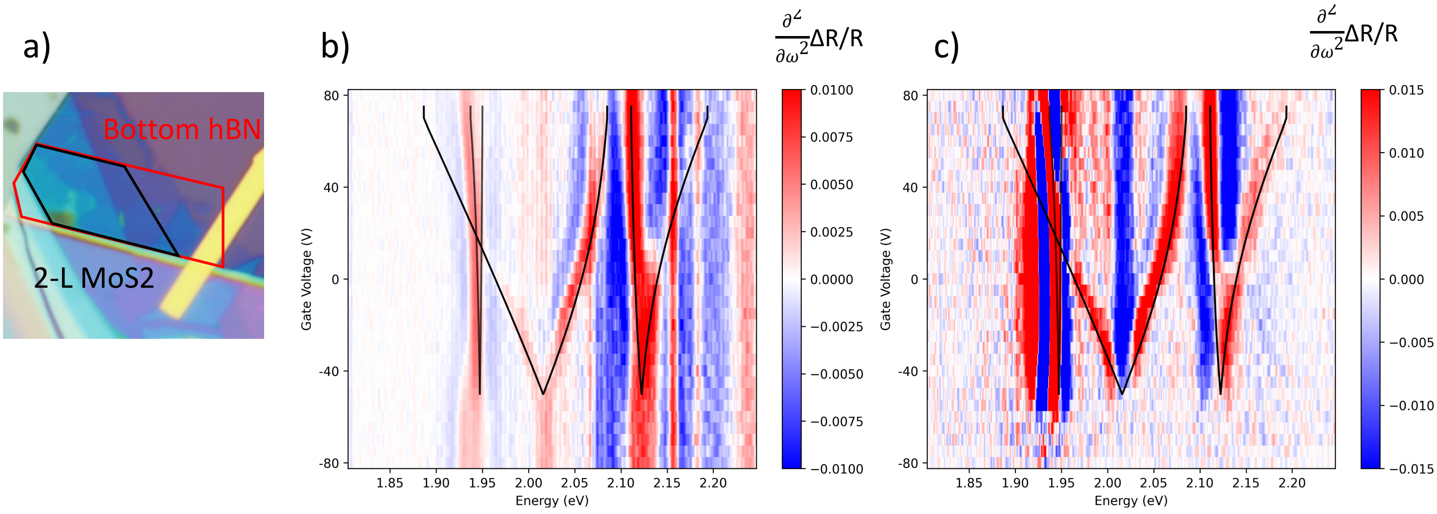

SI figure 9. Additional data from F_4TCNQ/2L-MoS_2 device #4: (a) Microscope image of device #4 2L-MoS_2. **(b)** Second derivative of reflectivity contrast. **(c)** Same map, normalized to the spectrum at -80 V for better visibility. Solid black line shows the positions of corresponding peaks in the device #1, taken from the coupling model. While the quality of the sample 2 is worse compared to sample 1, we observe very similar field-dependent behavior of X_A, X_B, and IX_1. The doping from the charge states on the interfaces is smaller, and IX_1 starts to split at -50 V, shifted by 30 V compared to the device #1.

Supplementary note S5: Simulation of maximum electric field for different molecules and TMD bilayers combinations

To calculate limiting electric fields for different combinations of TMDs and molecules, we numerically solve Eqs. (1-4), and extract the highest electric field. TMD parameters are taken from Ref. [3,7,12,13]. Molecule parameters are taken from Ref. [14–16] and summarized in SI table 3. To estimate the capacitance of different molecular layers, we use Eq. (6). In this case $C_{\mathrm{mol}} = \sigma_T^{max}/(E_{\mathrm{cond}} - E_{\mathrm{mol}})$. Where, E_{cond} is MoS_2 conduction band position 4.37 eV, and σ_T^{max} is the maximum charge transfer density to MoS_2 in referenced papers. The numbers used are $\sigma_T^{max} = 1 \cdot 10^{13}\ \mathrm{cm}^{-2}$ for Benzyl Viologen[14], and $\sigma_T^{max} = 5.5 \cdot 10^{13}\ \mathrm{cm}^{-2}$ for single sided Me-OED layer[15]. For CN6-CP we assume the same capacitance as calculated for F_4TCNQ, with LUMO energy at 5.94 eV (SI fig. 12). Applying these molecules to other TMDs that are not MoS_2, we assume capacitance to remain the same, while taking in account different bands offset.

	F_4TCNQ	CN6-CP	BV^0	Me-OED
E_{mol} (meV)	5300	5940	3583	3463
C_{mol} ($\mathrm{mF/m^2}$)	17.9	17.9	20.3	96.6

SI table 3. Molecules parameters used in the simulations. E_{mol} is the energy potential of molecular layer. For p-dopants it's equal to LUMO energy, for n-dopants – HOMO energy.

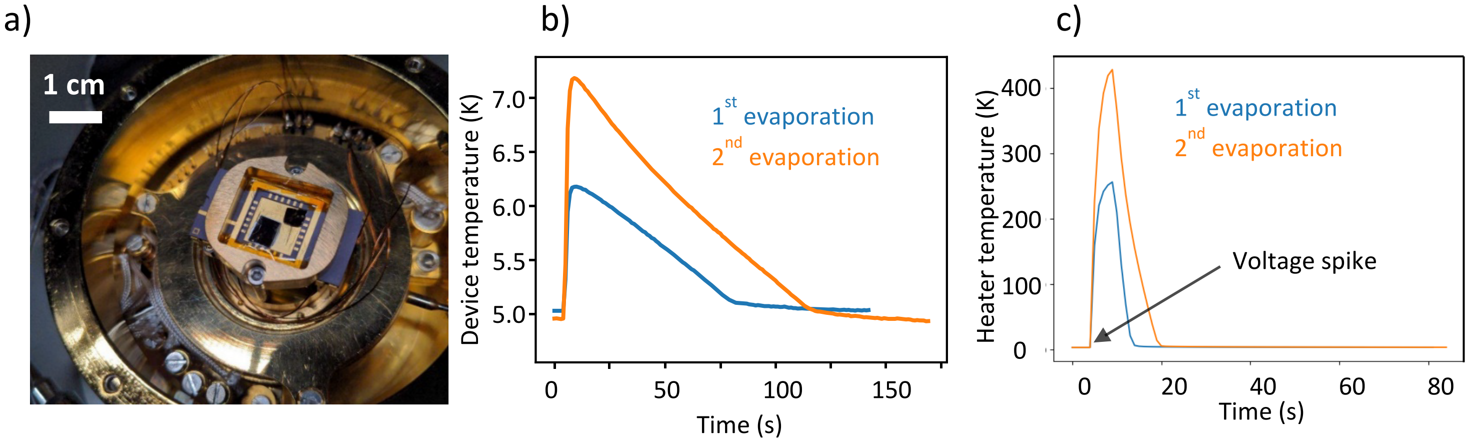

SI figure 10. In situ evaporation technique details (a) Photograph of the sample and evaporator chips loaded into the cryostat (the schematic of the same devices is shown in main text Fig. 1b). The chip carrier in the center is surrounded by the radiation shield of the cryostat. Additionally, in order to protect the inner surfaces of the cryostat from evaporated molecules the cell is covered by either thin (170 um) cover glass or a SiN membrane (500nm, Norcada). On the photo, this top cover is removed for loading purposes. **(b,c)** Temperature measured on the sample **(b)** and on the evaporator **(c)** during two consecutive evaporations using the in situ techniques described in the text. Both measurements done by monitoring the resistance of the gold stripe on each chip, similar to Ref. [17]. The evaporator chip does not have a good thermal contact with the chip carrier, and almost does not heat up the sample directly.

Supplementary note S6: General Working Methods

Reagents and Solvents: All reagents and solvents were used as purchased from commercial suppliers unless otherwise noted. The dry solvent THF was taken from the solvent purification system MB-SPS-800 (Braun). MeCN was dried over NaH dispersion for 10 min and subsequently quickly distilled under argon and stored over molecular sieves (3 Å) under exclusion of light.

^{1}H and ^{13}C NMR spectra were acquired on a JEOL ECZ 600 (600 MHz) or a Jeol ECZ600 S (600 MHz, solid state NMR) and analysed with MestReNova (version 14.2.3-29241). Chemical shifts (δ) are reported in parts per million (ppm) relative to the deuterated solvent and coupling constants (J) are represented in hertz (Hz). DMSO-d_6 is used as deuterated solvens and the residual solvent signals (DMSO-d_6: 2.50 ppm in ^{1}H NMR and 39.52 ppm in ^{13}C NMR) were used as reference in the ^{1}H NMR and ^{13}C NMR acquisition. The multiplicities of the signals are described using the following abbreviations: s = singlet, d = doublet, t = triplet, q = quartet, quint = quintet and br = broad.

High resolution mass spectra were obtained on an ESI-FTICR-MS: Ionspec QFT-7 (Agilent/Varian).

Elementar alanysis was done with a Elementar Vaörio EL (3 columns, C-H-N-S).

FT-Raman was measured by MultiRAM (100 mW with a wavelength of 1064 nm) from Bruker or an 532 nm XploRA Plus Raman Microscope from Horiba.

FT-IR were taken with a UATR Two from Perkin Elmer.

Cyclovoltomeric Measurements were performed with a Keithley 2450 Source Meter.

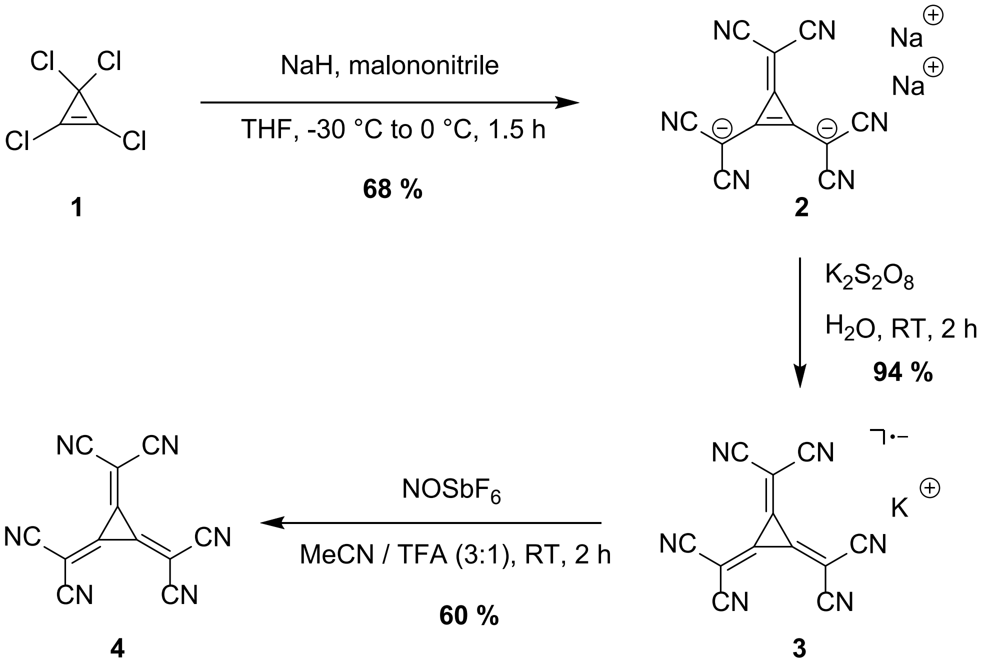

SI figure 11. CN6-CP synthesis. Schematic of preformed synthesis, similar to Refs. [18,19].

Supplementary note S7: Disodium 2,2',2''-(cyclopropane-1,2,3-triylidene)trimalononitrile (2)

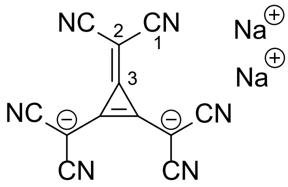

2

$C_{12}N_6Na_2$
MW: 274.15 g/mol

NaH (60 % dispersion in mineral oil, 3.56 g, 89.0 mmol, 6.33 eq.) was added to dry THF (100 mL) and cooled to -30 °C. Malononitrile (2.83 g, 42.9 mmol, 3.05 eq.) were added in small portions under strong gas evolution. The suspension was stirred for 15 min. At the same temperature, TCCP (**1**) (1.72 mL, 14.1 mmol, 1.0 eq.) in dry THF (10 mL) was added dropwise to the suspension. The solution was stirred for 30 min and was then allowed to warm to 0 °C over 1 h. The reaction turned from yellow to beige. Brine (10 mL) was added, and the reaction mixture was filtrated. The pale gray solid was recrystallized from water (150 mL) (for good crystallizations several attests were necessary). The precipitate was filtrated and washed with a small amount of ice-cold water. Drying yielded the product (**2**) (2.63 g, 9.59 mmol, 68 %) as pale gray, very thin, thread-like crystals.

^{13}C NMR (126 MHz, DMSO-d$_6$): δ[ppm] = 124.6 (C3), 121.1 (C1), 24.9 (C2) ppm.

^{1}H NMR (126 MHz, DMSO-d$_6$): no impurities found.

IR (FT): $\tilde{\nu}$ [cm^{-1}] = 2206, 2173, 1428, 1147, 566.

HRMS (ESI): m/z = (cal. $[C_{12}N_6Na]^-$ ($[M-Na]^-$): 251.0087; found 250.9594, (cal. $[C_{12}N_6]^-$ ($[M-2Na]^-$): 228.0189; found 227.9717, (cal. $[C_{12}N_6]^{2-}$ ($[M-2Na]^{2-}$): 114.0097; found 113.9759.

EA: ($C_{12}N_6Na_2$) C: 52.57%, N: 30.66%, H: 0.0%; found C: 52.60%, N 30.42%, H: 0.210%.

Raman: Raman shift (λ_{ex} 1064 nm) [cm^{-1}] = 2232, 2216, 2187, 2174 (CN), 1935, 1879 (CP_s), 1458, 1429 (CP_{as}), 1150.

Supplementary note S8: Potassium 2,2',2''-(cyclopropane-1,2,3-triylidene)trimalononitrile anioneradical (3)

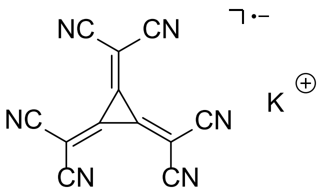

3

$C_{12}N_6K$
MW: 267.27 g/mol

$K_2S_2O_8$ (2.96 g, 10.9 mmol, 1.5 eq.) was dissolved in water (100 mL) and disodium 2,2',2''-(cyclopropane-1,2,3-triylidene)trimalononitrile (**2**) (2.00 g, 7.30 mmol, 1.0 eq.) was added in small portions. Immediately, a color change to blue/purple occurred. The reaction was stirred for 2 h at RT and bronze-colored small crystals formed. The suspension was filtered through a glass frit (P4), was washed with water (2 x 3 mL) and dried under reduced pressure. The product (**3**) (1,84 g, 6.88 mmol, 94 %) was obtained as purple bronze-shimmering-colored crystals.

^{13}C NMR (126 MHz, DMSO-d$_6$): δ [ppm] = no signals due to paramagnetism.

^{1}H NMR (126 MHz, DMSO-d$_6$): no impurities found.

IR (FT): $\tilde{\nu}$ [cm^{-1}] = 2928, 2213, 1483, 1469, 898.

HRMS (ESI): m/z = (cal. $[C_{12}N_6]^-$ ($[M-K]^-$): 228.0189; found 227.9768, (cal. $[C_{24}N_{12}K]^-$ ($[2M-K]^-$): 495.0011; found 494.9353.

Raman: Raman shift (λ_{ex} 532 nm) [cm^{-1}] = 1471 (CP$_{as}$), 1848 (CP$_s$), 2218 (CN).

EA: ($C_{12}N_6K$): C: 53.93%, N: 31.44%, H: 0.0%; found C: 53.61%, N: 31.40%, H: 0.92%.

Supplementary note S9: 2,2',2''-(cyclopropane-1,2,3-triylidene)trimalononitrile (4)

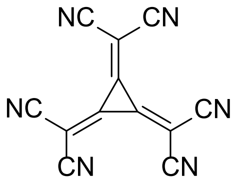

4

$C_{12}N_6$

MW: 228.17 g/mol

In an inert atmosphere, $NOSbF_6$ (273 mg, 0.935 mmol, 1.0 eq.) was dissolved in MeCN (6 mL) and TFA (2 mL). The radical anion $KC_6(CN)_6$ (250 mg, 1.03 mmol, 1.1 eq.) was added. A yellow-brown suspension formed and was stirred for 2 h. Afterwards, the precipitate was filtrated under argon, washed with TFA (2 x 1 mL) and dried under reduced pressure. A pale orange solid (127 mg, 0.556 mmol, 60 %) was obtained.

The compound either reacted with every solvent tested or was not soluble. Low levels of degradation can be observed over time by IR-spectroscopy at 3242 (sb), 1812, 1632 (sb) 1420 cm^{-1}. Samewise the colour changes from orange/yellow to brown over time. Storage under Argon at -30 °C helps to store the compound without darkening of the colour for a few weeks. Approaches with $Tl(III)(CO_2CF_3)_3$ as oxidation agent resulted in less pure product. Further purification was achieved by evaporation.

^{1}H NMR (300 MHz, solid): no signals found.

^{13}C NMR (300 MHz, solid): 135.5, sb 113.6, 85.1.

IR (FT): $\tilde{\nu}$ [cm^{-1}] = 2213, 1564, 1221, 1060, 608, 574.

IR (transmission, ZnSe): $\tilde{\nu}$ [cm^{-1}] = 2925, 2220, 1564, 1221, 1060, 608, 576.

HRMS (ESI): m/z = (cal. $[C_{12}N_6]^-$ ($[M-K]^-$): 228.02; found 228.00, (cal. $[C_{12}N_6MeO]^-$ ($[M+MeO]^-$): 259.13; found 259.13.

Raman: Raman shift (λ_{ex} 532 nm) [cm^{-1}] = 1098, 1472, 1736, 1768, 1846, 1808, 2228.

EA: ($C_{12}N_6$): C: 63.17%, N: 36.83%, H: 0.0%; found C: 56.49%, N: 33.26%, H: 2.09%.

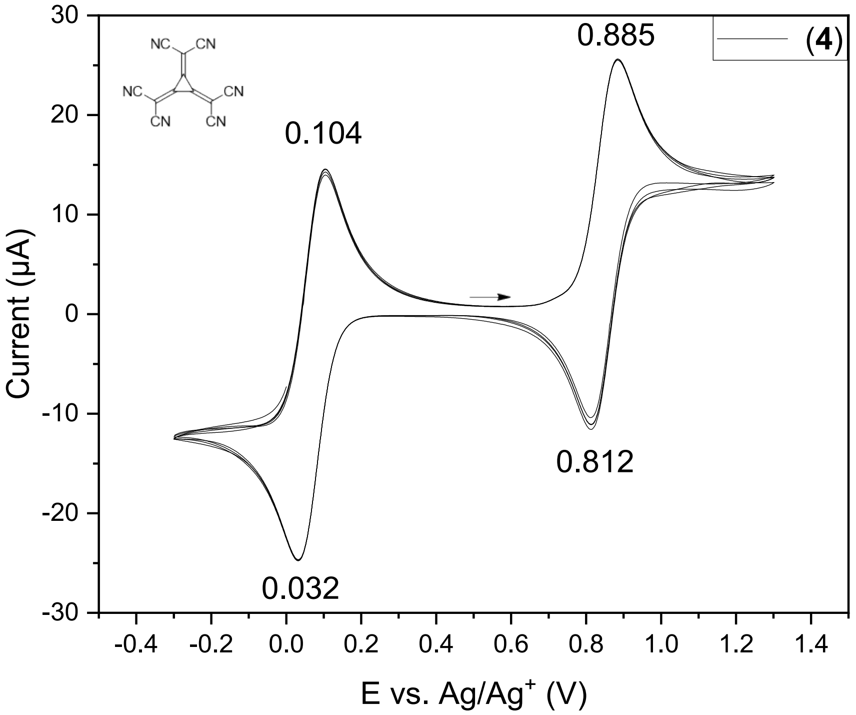

SI figure 12. **CV of CN6-CP**. Cyclic voltammetry of the molecule measured in dry and degassed in acetonitrile solutions with tetrabutylammonium hexafluorophosphate (0.1M) at 100 mVs^{-1} scan speed. As reference Ag/Ag^{+} was used, with a platin electrode and a platin counter electrode. The molecular concentrations were 10^{-3}. The second to fifth scan waves are depicted. A reduction potential of +0.85 versus Ag/Ag^{+} is equal to a LUMO energy of -5.94 eV.

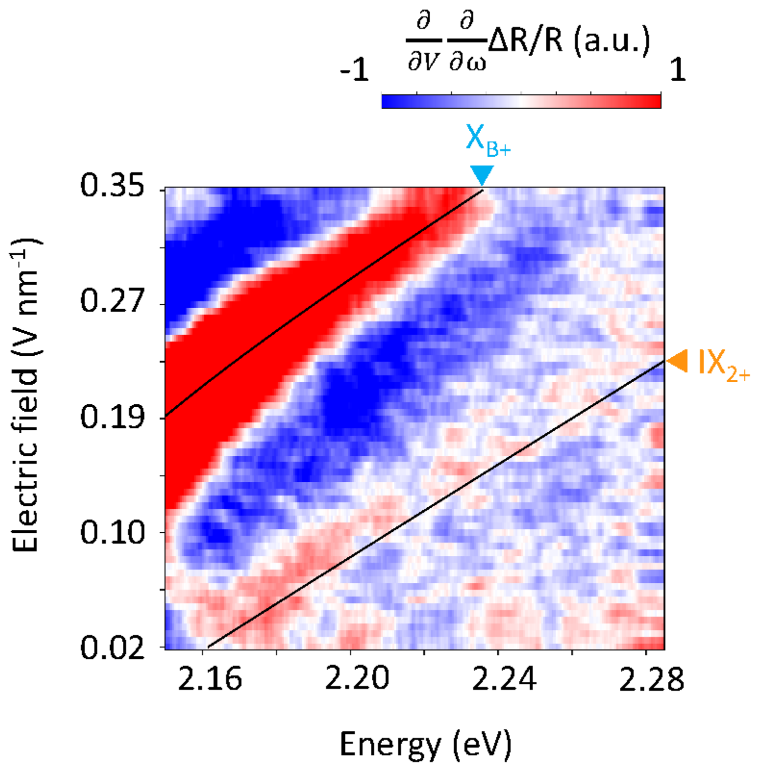

SI figure 13. Derivative of the reflectivity contrast with respect to V_G in the region of interest around IX_2, for device #2. Black lines are fitted positions of excitonic peaks, taken from the main text Fig. 2d.

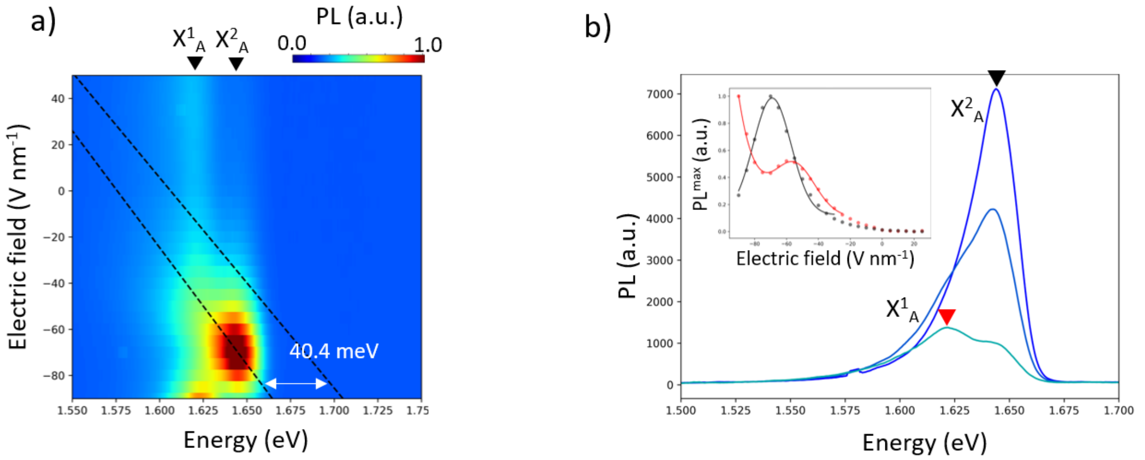

SI figure 14. **(a)** PL map for the MoSe2 – CN6CP device #5, that corresponds to the reflectivity map shown in Fig. 3b from the main text. Here we observe two excitonic peaks at 1.644 eV and 1.624 eV. Around the same electric field value as the avoided crossing in the Fig. 3b, both excitons brighten in PL as well. More importantly, X^1_A and X^2_A brighten at slightly different field values, consistent with the interlayer darks state, labeled IX_{dark}, crossing first X^2_A and then X^1_A. **(b)** PL spectra for three characteristic field values, where: X^1_A is brightest, X^2_A is brightest and an intermediate state. The inset shows fitted progression of X^1_A and X^2_A oscillator strengths with respect to electric field. These fits allow us to better extract the field values where IX_{dark} crosses X^1_A and X^2_A.

Supplementary note S10: Dipole moment definition

The dipole moment cited in the main text and SI table 2 corresponds to the interlayer exciton unperturbed by the interaction with its intralayer counterpart – in which case the dipole moment is close to the layer separation. This unperturbed value of the dipole moment enters the interaction model, that we use in our work to fit avoided crossing behavior between interacting interlayer and intralayer excitons [10]. For example, to fit the avoided crossing between IX_1 and X_B, the model takes as an input the constant energy of X_B and linear shift of IX_1 with a fixed dipole moment (dashed lines in the main text fig. 2a):

$$E_{IX\pm}' = E_{IX}^{0'} \pm F_Z \times d_{BL}$$

E_X and E_{IX} are then fitted using SI Eq. 9.

Another value for the dipole moment, commonly reported in the literature, is the linear fit to the Stark field splitting in the low electric field regime. In this case intralayer – interlayer interactions are ignored. Appling similar analysis to our data, we can obtain a different value for the dipole moment:

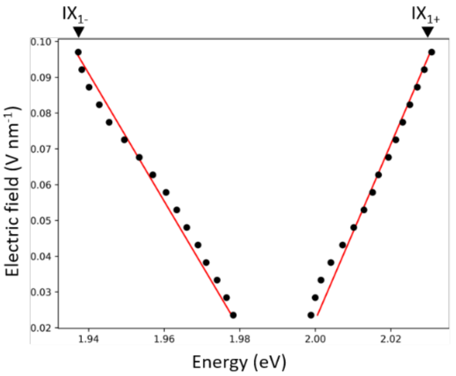

SI figure 15. Linear fits (red lines) for IX_{1-} and IX_{1+} excitons positions (black dots) at low electric field regime for device #2.

Corresponding comparison table:

Ref	IX_{1-} (e·nm)	IX_{1+} (e·nm)
Lorchat et al., Phys. Rev. Lett. 126, 037401 (2021)	0.48 ± 0.1	0.48 ± 0.1
Peimyoo et al., Nature Nanotech. 16, 888893 (2021)	≈ 0.4	≈ 0.4
Leisgang et al., Nature Nanotech. 15, 901907 (2020)	0.47 ± 0.01	0.39 ± 0.01
Zhao et al., Phys. Rev. B 105, L041411 (2022)	0.56 ± 0.01	0.56 ± 0.01
This work	0.55 ± 0.01	0.41 ± 0.01

SI table 4. Comparison of the extracted dipole moment between different publications, using the method of linear fitting at low electric fields.

	Structure	Used in
Device #1	$F_4TCNQ/2L\text{-}MoS_2$	Fig. 1d, e, SI Fig. 1, 2, 3, 4, 5, 6
Device #2	$CN6\text{-}CP/2L\text{-}MoS_2$	Fig. 2, SI Fig. 3, 5, 5.2, 8, 13,
Device #3	$F_4TCNQ/2L\text{-}MoSe_2$	Fig. 3a, SI Fig. 2, 5, 7,
Device #4	$F_4TCNQ/2L\text{-}MoS_2$	SI Fig. 9
Device #5	$CN6\text{-}CP/2L\text{-}MoSe_2$	Fig. 3b, SI Fig. 14

SI table 5. Devices overview. Devices numbers referenced throughout the main text and SI, corresponding TMD and molecule combination and figures where the data for these devices is shown.

1. Pisoni, R. *et al.* Absence of interlayer tunnel coupling of K-valley electrons in bilayer MoS2. *Physical Review Letters* **123**, 117702 (2019).

2. Maand, N. & Jena, D. Carrier statistics and quantum capacitance effects on mobility extraction in two-dimensional crystal semiconductor field-effect transistors. *2D Materials* **2**, 015003 (2015).

3. Hagel, J., Brem, S., Linderälv, C., Erhart, P. & Malic, E. Exciton landscape in van der Waals heterostructures. *Phys. Rev. Research* **3**, 043217 (2021).

4. Scholes, D. T. *et al.* The Effects of Crystallinity on Charge Transport and the Structure of Sequentially Processed F4TCNQ-Doped Conjugated Polymer Films. *Advanced Functional Materials* **27**, 1702654 (2017).

5. Stanfield, D. A., Mehmedović, Z. & Schwartz, B. J. Vibrational Stark Effect Mapping of Polaron Delocalization in Chemically Doped Conjugated Polymers. *Chem. Mater.* **33**, 8489–8500 (2021).

6. Wang, J. *et al.* Charge Transfer within the F $_4$ TCNQ-MoS $_2$ van der Waals Interface: Toward Electrical Properties Tuning and Gas Sensing Application. *Advanced Functional Materials* **28**, 1806244 (2018).

7. Laturia, A., Van de Put, M. L. & Vandenberghe, W. G. Dielectric properties of hexagonal boron nitride and transition metal dichalcogenides: from monolayer to bulk. *npj 2D Mater Appl* **2**, 1–7 (2018).

8. Peimyoo, N. *et al.* Electrical tuning of optically active interlayer excitons in bilayer MoS2. *Nature Nanotechnology* **16**, 888–893 (2021).

9. Leisgang, N. *et al.* Giant Stark splitting of an exciton in bilayer MoS2. *Nature Nanotechnology* **15**, 901–907 (2020).

10. Lorchat, E. *et al.* Supplementary Materials : Excitons in Bilayer MoS 2 displaying a collosal electric eld splitting and tunable magnetic response. *Physical Review Letters* **126**, 37401 (2021).

11. Sponfeldner, L. *et al.* Capacitively-coupled and inductively-coupled excitons in bilayer MoS$_2$. (2021).

12. Wang, Z., Chiu, Y. H., Honz, K., Mak, K. F. & Shan, J. Electrical Tuning of Interlayer Exciton Gases in WSe2 Bilayers. *Nano Letters* **18**, 137–143 (2018).

13. Das, S. *et al.* Highly Tunable Layered Exciton in Bilayer WS2: Linear Quantum Confined Stark Effect versus Electrostatic Doping. *ACS Photonics* **7**, 3386–3393 (2020).

14. Kiriya, D., Tosun, M., Zhao, P., Kang, J. S. & Javey, A. Air-Stable Surface Charge Transfer Doping of MoS2 by Benzyl Viologen. *J. Am. Chem. Soc.* **136**, 7853–7856 (2014).

15. Reed-Lingenfelter, S. N. *et al.* Compact Super Electron-Donor to Monolayer MoS2. *Nano Lett.* **22**, 4501–4508 (2022).

16. Park, S. *et al.* Temperature-Dependent Electronic Ground-State Charge Transfer in van der Waals Heterostructures. *Advanced Materials* **33**, 2008677 (2021).

17. Greben, K., Arora, S., Harats, M. G. & Bolotin, K. I. Intrinsic and Extrinsic Defect-Related Excitons in TMDCs. *Nano Letters* **20**, 2544–2550 (2020).

18. Fukunaga, T., Gordon, M. D. & Krusic, P. J. Negatively substituted trimethylenecyclopropanes and their radical anions. *J. Am. Chem. Soc.* **98**, 611–613 (1976).

19. Karpov, Y. *et al.* High Conductivity in Molecularly p-Doped Diketopyrrolopyrrole-Based Polymer: The Impact of a High Dopant Strength and Good Structural Order. *Advanced Materials* **28**, 6003–6010 (2016).
